# Why Medical Students Should Be Trained in Disaster Management: Our Experience of the Kashmir Earthquake

**DOI:** 10.1371/journal.pmed.0030382

**Published:** 2006-09-26

**Authors:** Ahmad Ayaz Sabri, Muhammad Ahad Qayyum

## Abstract

Two medical students say that they were unprepared for the task of treating casualties of the Kashmir earthquake, since they had not had any training in disaster management.

During the past 20 years, natural disasters have claimed more than 3 million lives worldwide, affected at least 800 million people, and resulted in property damage exceeding $US50 billion [1]. On 8 October 2005, an earthquake measuring 7.6 on the Richter scale (categorized as a major quake) struck Pakistan, along with parts of India and Afghanistan. [2]. The epicenter was about 19 kilometers northeast of Muzaffarabad (the capital of Pakistan-administered Kashmir), and 100 kilometers north-northeast of Islamabad (the capital of Pakistan) [2]. In addition to causing direct casualties (Table 1), the earthquake resulted in widespread damage, wiping out entire villages and flattening towns and cities in Pakistan-administered Kashmir and the North West Frontier Province (NWFP) of Pakistan. About 60 percent of concrete buildings in Muzaffarabad collapsed, and an estimated 3.3 million people were rendered homeless [2]. The quake triggered landslides, burying entire villages and roads in many areas of NWFP and Pakistan-administered Kashmir [2]. In this essay, we discuss the role that medical students played in disaster relief activities.

**Table 1:**
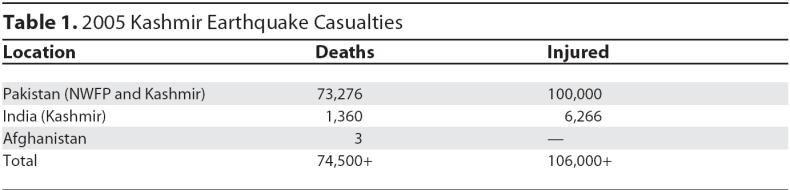
2005 Kashmir Earthquake Casualties

## Rescue and Relief

**Figure 1:**
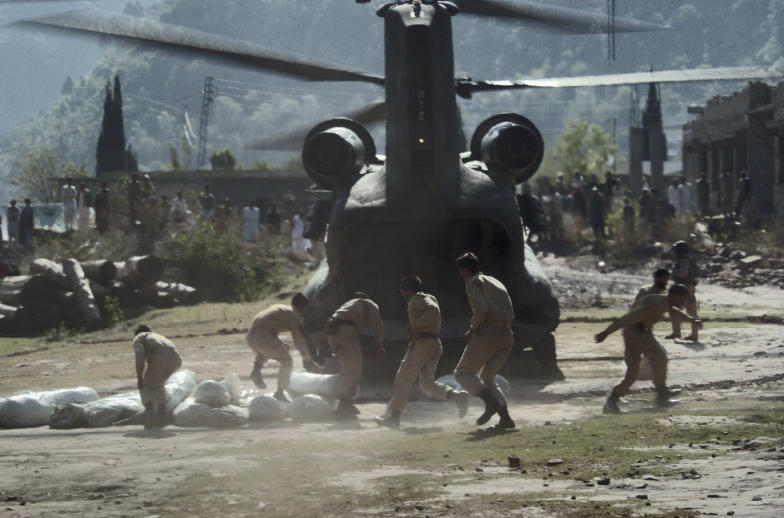
Pakistani Soldiers Unload Tents to Deliver to People Affected by the Kashmir Earthquake (Photo: Timothy Smith, United States Navy)

Immediately after the earthquake occurred, the largest rescue and relief operation was launched in the history of Pakistan. The Pakistani Army was directed to extend help to the civilian population in the quake-affected areas (Figure 1), and all civilian and military hospitals were directed to deal with the situation on an emergency basis. Many countries, international organizations, and nongovernmental organizations offered relief aid to the region in the form of donations as well as relief supplies, including food, medical supplies, tents, and blankets [3]. International rescue and relief workers brought rescue equipment, including helicopters and rescue dogs.

## Challenges for Medical Students at the Disaster Scene

Hospitals across Pakistan sent teams of health professionals: general surgeons, orthopedic surgeons, physicians, pediatricians, paramedics, and senior (final-year) medical students. As final-year medical students, our job is usually to observe and assist the senior doctors in their respective wards and emergency rooms. However, we were entirely unprepared for the task of treating casualties of the Kashmir earthquake—we had not had any disaster management training or exposure to real-time emergency care situations.

We were sent by our hospital and college, under the supervision of senior doctors, to the quake-struck area of Kashmir. When we reached the area, it looked like a war zone, with injured people everywhere awaiting help. Most of the buildings we saw had collapsed or were on the verge of collapsing. As young medical students, we had never seen a disaster of this massive scale. We were not expecting to carry out tasks without the facilities and technologies that we were used to working with in emergency rooms. The situation was beyond our imagination. We were in a mountainous area and almost all of the roads had vanished due to landslides and boulders, leaving thousands of people trapped in the valleys. These people could be rescued only by air or by foot after a ten- to 12-hour hike. Physically strong and willing medical students were sent as small teams along with military personnel to these valleys for search and rescue.

Almost all of these students had to work without supervision, because elderly and physically weak senior doctors were not able to undertake the long hike. Being unsupervised while caring for patients was a real test for us as semi-trained medical students—we were used to working in hospitals and obeying the orders of senior doctors, and we had no training in making crucial decisions in emergency situations. As medical students placed in this demanding situation, we faced a number of difficult challenges.

**Keeping our emotions in check.** The initial challenge for us was to keep our emotions under control and to try and remain calm and act rationally. Some people were calling for help from the rubble and others were lying out in the open, exposed to the cold weather, waiting for medical aid. We were extremely confused as to whether we should try to save those who were in the rubble or to treat those who were lying outside. Many students were overwhelmed with emotion at the sight of seeing people trapped in the rubble of semi-collapsed buildings, and so rushed to pull people out, without realizing the frail nature of the buildings. Some students were seriously injured as the partially collapsed buildings fell on them due to aftershocks. Instead of helping others, some of us became a burden on the other rescuers and hampered the rescue efforts.

**Prioritizing medical attention.** The next most daunting challenge was to prioritize care for the large number of people who required medical attention. We saw a variety of injuries, including limb, head, neck, chest, and abdominal injuries. However, when we started to treat the injured, we had no training in whether to treat, for example, a cervical spine injury or a head injury first. We kept on treating patients without any sense of prioritizing who needed care first. We realize this is poor medical practice, but we had no choice.

As we treated people, our hands shook. In urban teaching hospitals, doctors and senior medical students are used to working with highly advanced technology and completely depend upon sophisticated investigations for treating patients in emergency rooms. The problem was not our level of knowledge; rather, it was the environment we were working in. Buildings were leveled. Children were screaming in pain. Where once a village of 5,000 people thrived, all that remained was an open field and debris of what used to be people's property. We had to provide emergency relief out in the open, exposed to the elements, without any facilities, infrastructure, or guidance from our seniors.

**Treating injured children.** The earthquake struck at 08:50 Pakistan Standard Time [2], and so most of those affected by falling buildings were women and children, since most of the men in that area had gone to work in the open fields. Treatment of injured children was another challenge for which we had never been prepared. During our routine training in medical school we had not received specific training in managing children's injuries.

**Gender issues.** Most of the affected population belonged to traditional, conservative communities, where men are not allowed to treat female patients. A major obstacle for us was to convince the men to let us treat the seriously injured females; otherwise they could lose their lives or limbs. Communication skills played a crucial role, but we believe that we could have communicated more effectively if we had received prior training.

## Training Health-Care Students in Disaster Management

Disaster management is an essential component of medical training, but unfortunately this component is largely missing from medical and nursing curricula. Our experience shows how a lack of training in disaster management can have unfortunate consequences for both patients and health-care students. We believe that in countries faced with the risk of natural disasters, a risk that is likely to increase due to global warming [4], the training of health professionals should be designed with an emphasis on regional disaster management.

## Abbreviations

NWFP: North West Frontier Province
